# Patterns of Physical Activity Among Overweight and Obese Adults

**Published:** 2009-06-15

**Authors:** Deborah Rohm Young, Gerald J. Jerome, Chuhe Chen, Daniel Laferriere, William M. Vollmer

**Affiliations:** Department of Epidemiology and Biostatistics, School of Public Health, University of Maryland; Towson University and Johns Hopkins University, Baltimore, Maryland; Kaiser Permanente Center for Health Research, Portland, Oregon; Kaiser Permanente Center for Health Research, Portland, Oregon; Kaiser Permanente Center for Health Research, Portland, Oregon

## Abstract

**Introduction:**

Little is known about patterns of physical activity in overweight and obese adults, although they are at high risk for chronic disease and can benefit from physical activity. We describe patterns of moderate-to-vigorous physical activity (MVPA) and MVPA in bouts of 10 minutes or longer in overweight and obese adults.

**Methods:**

Overweight and obese participants (n = 1,648) who were screened for the multicenter Weight Loss Maintenance Trial wore RT3 accelerometers for at least 3 weekdays and 1 weekend day. We determined minutes spent in moderate physical activity, vigorous physical activity, and MVPA overall, by weekday vs weekend, and by time of day. We also measured bouts of at least 10 minutes of sustained MVPA.

**Results:**

Participants were active for an average of 15.8 minutes per day. Among those who engaged in bouts of MVPA, the average bout was 33.3 minutes long. Participants who were younger than 50 years, male, non-African American, or overweight were more active than were those who were older than 50, female, African American, or obese. Participants were more active on weekends than on weekdays and in the morning than in the afternoon or evening. Only 2% of participants were active for 60 or more minutes per day.

**Conclusion:**

We found differences in physical activity patterns by demographic characteristics, day, and time of day. Weekend mornings may be an opportune time to promote additional physical activity.

## Introduction

The high prevalence of overweight and obesity affects Americans' health and the economic well-being of the United States. Approximately two-thirds of American adults are either overweight or obese (1). Excess weight increases risk for cardiovascular diseases, cancer, and premature death (2). A recent analysis indicates that obesity accounted for more than 100,000 excess deaths in 2000 (3). The economic cost of obesity in 1995 was $99.2 billion, representing 5.7% of US health expenditures for that year (4).

Physical activity contributes to weight loss and maintenance. Although several groups recommend 30 minutes of daily moderate physical activity (MPA) (5,6), more may be needed to promote weight loss and weight maintenance. An Institute of Medicine report recommends at least 60 minutes of daily MPA to maintain a healthy weight (7). Although researchers know that overweight and obese people exercise less than do their healthy-weight peers (8), little is known about the level of activity and how it compares with recommendations.

Physical activity is typically assessed from self-report. Recent technological advances have made accelerometry, a way to objectively assess physical activity, available for large-scale research trials. Objective measures overcome many of the limitations of self-report instruments, particularly measurement error associated with recall (9). In addition, obese people may overreport physical activity (10), which increases recall bias. Accelerometers can more accurately assess intensity, duration, and daily patterns of physical activity without relying on error-prone recall methods.

We used accelerometers to measure baseline physical activity in a large sample of overweight and obese people who were screened for participation in the Weight Loss Maintenance Trial. We measured total minutes of MPA, vigorous physical activity (VPA), and moderate-to-vigorous physical activity (MVPA) and activity performed in bouts of at least 10 minutes (the minimum amount associated with health benefits [6]). This information is needed to more effectively promote physical activity among overweight and obese people, who are at excess risk for chronic disease and premature death.

## Methods

### Sample

The Weight Loss Maintenance Trial was a randomized clinical trial designed to compare the effects of 2 interventions on sustaining long-term weight loss after initial weight loss (11,12). The trial was conducted from 2003 through 2007 at clinical centers in Baltimore, Maryland; Baton Rouge, Louisiana; Durham, North Carolina; and Portland, Oregon. Study participants were aged 25 years or older, had a body mass index (BMI) 25 to 45 kg/m^2^, and were at high risk for developing cardiovascular disease (taking antihypertensive or cholesterol-lowering medication). Before randomization, eligible people underwent a 5-month intensive weight loss intervention, and those who lost at least 4 kg were invited to participate in the Weight Loss Maintenance Trial. All participants provided informed consent in accordance with participating clinical centers' institutional review boards. We describe screening data from participants who met eligibility criteria and entered the 5-month weight loss intervention (n = 1,685). All data were collected before beginning the initial weight loss program.

### Assessment of physical activity

We assessed physical activity by using RT3 (Stayhealthy, Inc, Monrovia, California), a triaxial accelerometer that provides an objective measure of physical activity. The instrument detects acceleration from vertical, horizontal, and anterior-posterior planes and converts the information into "counts." More acceleration results in higher counts in a specified interval (typically 1 minute). The instrument has adequate intrainstrument and interinstrument coefficients of variation at the higher hertz frequencies (coefficients of variation <10% and <2.5%, respectively) when tested on a vibration table (13). The correlations between accelerometer counts and submaximal oxygen uptake for treadmill walking at different speeds and nonregulated physical activity are 0.79 and 0.89, respectively (14). Energy expenditure determined from the RT3 and doubly labeled water is significantly correlated in overweight and obese adults (*r* = 0.55) (15).

During a screening visit, clinic staff gave participants an RT3 that was programmed to capture data in 1-minute increments. They showed participants how to place the accelerometer above the right hip and asked them to wear the monitor during all waking hours (excluding time spent swimming and showering) for 7 days. Participants were instructed that the minimum acceptable wear time was 10 hours per day for 4 days, which had to include at least 1 weekend day. Participants returned the monitor to the clinic, where data were downloaded and assessed. Participants who did not wear the accelerometer for the minimum time were asked to wear the monitor again. If the second measurement period did not meet the minimum wear time, we processed the data record with the longest wear time. We required a minimum of 6 hours of wear time per day for 4 days, including at least 1 weekend day, for participants to be included in our analysis.

Accelerometry data were processed according to methods described elsewhere (16). To remove potential spurious movement related to placing the monitor, the first and last 5 minutes were removed from each data file. We assumed that single nonzero minutes surrounded by zero minutes were spurious counts, and we reset them to zero. We assumed that accelerometers were not worn for periods of zero activity that lasted at least 15 minutes. We used previously defined cutpoints (14) to define minutes of MPA (1,316.6-2,636.5 counts/min) and VPA (>2,636.5 counts/min). We combined these categories into MVPA.

### Assessment of BMI and demographic variables

We measured height by using a wall-mounted stadiometer and weight by using a calibrated scale. We defined overweight as BMI 25.0 to 29.9 kg/m^2^ and obese as BMI 30.0 kg/m^2^ or higher. We administered questionnaires to assess sex, age, and race. Because most non-African Americans were white and to be consistent with prior research (12), we classified race as African American or non-African American.

### Statistical analysis

We computed total minutes of MPA, VPA, and MVPA and minutes of MVPA that occurred in bouts of at least 10 minutes (bout minutes) for each day (16). We then estimated weekly physical activity as a weighted sum of mean weekday and mean weekend physical activity. Using these measures, we computed the percentage of participants who met recommendations of 30 minutes or more of MVPA on at least 5 days per week (operationalized as 150 minutes of MVPA in 7 days) (5,6). Because more stringent recommendations for maintaining weight loss suggest at least 60 minutes of MVPA per day (7), we also evaluated the proportion of participants who met this criterion (ie, at least 60 minutes of MVPA for each complete day of wearing the accelerometer).

To elucidate physical activity patterns, we separately examined data for weekdays vs weekends and by time of day. We defined morning as 6 am to noon, afternoon as noon to 6 pm, and evening as 6 pm to midnight. We also examined patterns of physical activity by selected demographic factors, including age, sex, race, and weight.

We used repeated-measures analysis of variance to determine the effects of age, sex, race, and weight on activity, while accounting for within-person day-to-day and within-day correlations. We determined the effects of weekend vs weekday and time of day on activity by using the same analytic procedures. Because of the expected redundancy of results when analyzing MVPA in segments (weekend vs weekday and time of day) by age, sex, race, and overweight status, we do not report the results of these analyses. For most analyses, we normalized total minutes of daily physical activity to a 12-hour day to equalize the different intervals participants actually wore the accelerometers; for example, if a participant wore the accelerometer for 10 hours and recorded 15 minutes of MVPA, this would be normalized to 18 minutes in a 12-hour day. For time-of-day analyses, we included only participants for whom data were available for at least 3 hours in a time interval (eg, mornings), and no normalization was done. We used SAS version 9.1 (SAS Institute, Inc, Cary, North Carolina) for all statistical analyses.

## Results

Of the 1,685 participants who were screened for inclusion in the Weight Loss Maintenance Trial, 1,648 (98%) wore their accelerometers for at least 6 hours per day and made up the study sample. Of the 1,685 who were screened, 82% wore their accelerometers for at least 10 hours per day. Mean age of participants was 60 years (standard deviation, 9 years). Two-thirds of participants were women, 43% were African American, and 79% were obese. Mean BMI was 28.2 kg/m^2^ among overweight participants and 35.9 kg/m^2^ among obese participants.

On average, participants were active for less than 16 minutes per day, and less than 3 of these minutes was spent in VPA (Table 1). Participants who were younger, male, non-African American, and overweight were significantly more active than were participants who were older, female, African American, and obese.

Although mean minutes of MVPA differed significantly by weekday vs weekend day, the magnitude of the difference was negligible (0.2 min) (Table 2). Overall, participants were more active in the mornings than in the afternoons and least active in the evenings. Although subgroup data were not tested for significance, this trend was observed for all demographic and weight status subgroups.

A total of 810 (49%) participants recorded at least 1 MVPA bout of at least 10 minutes during the monitoring period. These bouts lasted an average of 33.3 minutes (Table 3) and lasted significantly longer for men and overweight participants than for women and obese participants, although the difference by weight status was minimal (0.3 minutes). Bouts lasted approximately 5 minutes longer on weekend days than on weekdays and approximately 5 minutes longer in the morning than in the afternoon or evening. Participants were most active (in terms of total MVPA minutes as well as bout minutes) on weekend mornings; in comparison, participants recorded 7 to 8 fewer minutes of MVPA on weekday and weekend evenings.

When all MVPA minutes were considered, approximately one-fourth of participants met recommendations of 30 minutes of MVPA on 5 days per week (Table 4). Forty percent of men and 36% of overweight participants met the recommendation of at least 150 minutes of MVPA in 7 days. In contrast, only 18% of women and 23% of obese participants met this recommendation. Few participants (≈2%) met the more stringent target of at least 60 minutes of MVPA per day.

## Discussion

On average, our participants were active for less than 16 minutes per day. Almost half of participants had at least 1 bout of MVPA that lasted at least 10 minutes, and each bout, on average, exceeded 30 minutes. MVPA was most likely in the morning and on weekends, and mornings were times in which MVPA was most likely to occur.

Similar findings have been reported in national data sets that used self-report instruments (17,18). A previous study used accelerometry data from the National Health and Nutrition Examination Survey (NHANES) and found similar age, sex, and race associations with physical activity, although results were not reported by weight status (19). Another study that used accelerometers showed that overweight adults were more physically active than were obese adults, but the sample size was small and mean differences were not significant (20). In our large, geographically diverse sample, obese adults were significantly less active than were their overweight peers. However, because our study design was cross-sectional, we cannot determine if obesity decreased physical activity or if lower physical activity over time contributed to obesity.

This study is among the first large-scale studies to objectively measure physical activity and examine activity levels across different times of day. In general, overweight and obese adults were more likely to be active in the morning, particularly on weekends. Adults aged 50 years or more were active 2 minutes less than were adults aged less than 50 years, and this finding supports previous findings that older adults are less active in the evening than are younger adults (21). Another study reported more activity on weekends than on weekdays (8), which is consistent with our results. These results suggest that studies of physical activity patterns should examine time of day and weekdays vs weekend.

Information from this study has implications for physical activity promotion. Overweight and obese adults were most active on weekends and mornings. Efforts to encourage additional physical activity at times when adults are most likely to be active should promote weekend mornings. Conversely, efforts that seek to encourage activity at times when baseline activity is low should promote weekday afternoons and evenings.

Because of the various methods in which physical activity data are collected and reported, making comparisons across studies is difficult. National self-report data suggest that approximately 50% of all adults (17) and 20% to 30% of overweight and obese adults (22,23) meet national recommendations. National data on minutes of MVPA are only available from NHANES accelerometry data. On the basis of the mean number of minutes reported, our sample was less active than was the general US population (19). Physical activity assessed with accelerometry can provide more precise information regarding MVPA minutes than is available from self-report.

NHANES accelerometry data indicate that only 3.5% of US adults meet physical activity recommendations (19). This percentage contrasts with the more than 25% of our participants who met recommendations. The difference is surprising because our participants were all overweight or obese, conditions associated with low physical activity. Methodological differences may explain the discrepancy. In the NHANES data, investigators only considered bouts of at least 10 minutes, which had to total 30 minutes per day on 5 days per week. We counted all minutes of MVPA and considered 150 minutes in 7 days to meet recommendations. Given that less than half of our sample engaged in even 1 bout of at least 10 minutes, if we had used more conservative criteria for meeting recommendations, our findings probably would have reflected those of the NHANES data.

Few participants met the more stringent recommendation of at least 60 minutes of physical activity per day. A recent study (8) reported that 13% of overweight and obese participants met this recommendation, but that sample was smaller than ours (n = 62), and participants were specifically recruited into a study that evaluated exercise and energy expenditure, which may have attracted more active participants.

The number of minutes of physical activity determined by accelerometry depends on the cutpoints used, which could overestimate or underestimate true physical activity levels. Only 1 validation study used the RT3 to assess accelerometry counts by oxygen uptake (14). Additional validation studies are needed to determine appropriate cutpoints for light, moderate, and vigorous physical activity for instruments such as the RT3.

Physical activity data are often skewed, and this was the case with our data. We considered presenting the data as medians and interquartile ranges rather than as means and standard deviations. However, other studies of physical activity that used accelerometry reported means (8,19), and we chose this approach to be consistent with other published work.

Strengths of our study are a large sample size, the use of accelerometry data, high adherence with wearing the accelerometer (98%), diverse demographic characteristics of the sample, and our ability to examine daily and weekly patterns of physical activity. Although NHANES collected accelerometer data from 2002 through 2004 (19), this is the first report that focuses solely on overweight and obese adults. The major limitation of these data is that participants were enrolling in a weight loss trial, and these findings may not be entirely generalizable to the US overweight and obese population.

We found that previously reported differences in physical activity by demographic features and weight status persist when physical activity is measured with an objective method. We also found that obese people are physically active, although not at recommended levels. Weekend mornings, a time when our participants were more active, may be an opportune time to promote additional physical activity, particularly in bouts of 10 minutes or longer, to meet national recommendations.

## Figures and Tables

**Table 1:** Baseline Physical Activity Level of Participants Screened for Inclusion in the Weight Loss Maintenance Trial

Variable	Mean (SD) Minutes of Daily Physical Activity^a^		

	MPA	VPA	MVPA
Total sample (n = 1,648)	13.6 (18.0)	2.2 (8.0)	15.8 (22.5)
**Age, y (*P* < .001)^b^**			
<50 (n = 454)	15.0 (17.1)	2.4 (7.5)	17.3 (21.1)
≥50 (n = 1,194)	13.1 (18.3)	2.1 (8.2)	15.3 (23.0)
**Sex (*P* < .001)^b^**			
Male (n = 550)	18.8 (23.0)	3.6 (10.8)	22.4 (29.6)
Female (n = 1,098)	11.0 (14.1)	1.5 (6.0)	12.5 (16.9)
**Race (*P* = .002)^b^**			
Non-African American (n = 946)	14.7 (19.7)	2.5 (9.2)	17.2 (24.8)^b^
African American (n = 702)	12.2 (15.3)	1.8 (6.0)	13.9 (18.8)
**Weight status (*P* < .001)^b^**			
Overweight (n = 351)	16.7 (20.1)	3.0 (9.2)	19.6 (24.6)
Obese (n = 1,297)	12.8 (17.3)	2.0 (7.6)	14.8 (21.8)

Abbreviations: SD, standard deviation; MPA, moderate physical activity; VPA, vigorous physical activity; MVPA, moderate-to-vigorous physical activity.

a Minutes of physical activity were recorded on an accelerometer worn for ≥6 hours/day and extrapolated to a 12-hour day. See the "Methods" section for definitions of MVA, VPA, and MVPA.

b Repeated-measures analysis of variance adjusted for clinical site and other covariates in table; *P* values calculated only for between-group differences in MVPA.

**Table 2:** Baseline Physical Activity Level of Participants Screened for Inclusion in the Weight Loss Maintenance Trial, by Weekday vs Weekend Day and Time of Day

Variable	Mean (SD) Minutes of Daily MVPA^a^				

	Weekday vs Weekend Day		Time of Day		

	Weekday	Weekend	Morning	Afternoon	Evening
Total sample (n = 1,648)^b^	15.8 (20.9)	16.0 (25.9)	8.3 (15.3)	7.6 (13.8)	3.1 (8.3)
**Age, y**					
<50 (n = 454)	17.4 (19.7)	17.2 (24.2)	8.2 (13.8)	8.5 (12.8)	4.4 (10.0)
≥50 (n = 1,194)	15.2 (21.4)	15.5 (26.5)	8.4 (15.9)	7.3 (14.2)	2.6 (7.5)
**Sex**					
Male (n = 550)	21.8 (27.0)	23.9 (34.9)	11.3 (19.2)	11.2 (19.2)	4.0 (9.5)
Female (n = 1,098)	12.7 (16.3)	12.0 (18.5)	6.8 (12.6)	5.7 (9.6)	2.7 (7.5)
**Race**					
Non-African American (n = 946)	16.7 (22.5)	18.4 (29.5)	8.7 (16.3)	8.4 (15.6)	3.3 (8.9)
African American (n = 702)	14.5 (18.6)	12.7 (19.3)	7.8 (13.8)	6.5 (10.9)	2.9 (7.5)
**Weight status**					
Overweight (n = 351)	19.3 (22.7)	20.5 (28.5)	10.6 (17.3)	8.9 (14.5)	4.3 (10.4)
Obese (n = 1,297)	14.8 (20.3)	14.8 (25.0)	7.7 (14.7)	7.2 (13.6)	2.8 (7.6)

Abbreviations: SD, standard deviation; MVPA, moderate-to-vigorous physical activity.

a Minutes of physical activity were recorded on an accelerometer worn for ≥6 hours/day and extrapolated to a 12-hour day for weekday vs weekend day analyses. See the "Methods" section for definition of MVPA.

b Repeated-measures analysis of variance *P* = .003 for weekday vs weekend day and *P* < .001 for time of day, adjusted for clinical site and other covariates in table. No other subgroup analyses were performed.

**Table 3:** Mean Length of Physical Activity Bouts of ≥10 Minutes Among Participants Screened for Inclusion in the Weight Loss Maintenance Trial Who Recorded ≥1 Such Bout, by Weekday vs Weekend Day and Time of Day

Variable	Mean (SD) Minutes of MVPA per Bout^a^					

	Overall (1,807 bouts)^c^	Weekday vs Weekend Day		Time of Day^b^		

		Weekday (1,291 bouts)	Weekend (516 bouts)	Morning (742 bouts)	Afternoon (721 bouts)	Evening (325 bouts)
Total (n = 810)^d^	33.3 (26.2)	31.8 (23.4)	36.9 (31.8)	34.3 (23.7)	28.6 (24.5)	28.9 (16.4)
**Age, y**						
<50 (n = 220)	31.9 (24.0)	29.7 (21.1)	37.7 (29.6)	32.0 (22.0)	26.5 (21.0)	29.0 (16.9)
≥50 (n = 590)	33.8 (26.9)	32.6 (24.2)	36.7 (32.5)	35.0 (24.2)	29.3 (25.5)	28.8 (16.1)
**Sex**						
Male (n = 348)	35.7 (31.7)	34.1 (27.5)	39.4 (39.5)	35.3 (26.9)	31.1 (30.4)	28.1 (15.8)
Female (n = 462)	31.3 (20.2)	30.1 (19.4)	34.6 (22.1)	33.6 (20.8)	25.9 (15.7)	29.5 (16.9)
**Race**						
Non-African American (n = 512)	33.9 (27.7)	32.1 (24.1)	38.2 (34.1)	33.9 (25.2)	29.6 (25.4)	29.7 (16.7)
African American (n = 298)	32.1 (23.1)	31.5 (22.1)	34.0 (25.7)	35.1 (21.0)	26.0 (21.8)	27.6 (15.9)
**Weight status**						
Overweight (n = 229)	33.5 (24.6)	32.0 (21.8)	37.2 (30.2)	36.0 (22.5)	28.2 (22.3)	30.3 (17.7)
Obese (n = 581)	33.2 (26.9)	31.8 (24.1)	36.8 (32.6)	33.5 (24.3)	28.7 (25.3)	28.2 (15.7)

Abbreviations: SD, standard deviation; MVPA, moderate-to-vigorous physical activity.

a Minutes of physical activity were recorded on an accelerometer worn for ≥6 hours/day and extrapolated to a 12-hour day for overall and weekday vs weekend day analyses. See the "Methods" section for definition of MVPA.

b 19 bouts were excluded because they crossed time periods.

c Repeated-measures analysis of variance found significant differences in overall bout minutes by sex and weight status (both *P* = .002), adjusted for clinical site and other covariates in table.

d Repeated-measures analysis of variance found significant differences for the total subsample by weekday vs weekend day and by time of day (both *P* < .001), adjusted for clinical site and other covariates in table. No other subgroup analyses were performed.

**Table 4:** Percentage of Participants Screened for Inclusion in the Weight Loss Maintenance Trial Who Met Different National Recommendations for Physical Activity

Variable	% of Participants	
	≥30 min MVPA/d on ≥5 d/wk^a^,^b^	≥60 min MVPA/d^c^
Overall (n = 1,648)	25.7	2.1
**Age, y**		
<50 (n = 454)	29.3	2.4
≥50 (n = 1,194)	24.3	1.9
**Sex**		
Male (n = 550)	40.2	4.6
Female (n = 1,098)	18.4	0.8
**Race**		
Non-African American (n = 946)	28.4	2.6
African American (n = 702)	21.9	1.3
**Weight status**		
Overweight (n = 351)	36.2	3.4
Obese (n = 1,297)	22.8	1.7

Abbreviation: MVPA, moderate-to-vigorous physical activity.

a Operationalized as ≥150 min MVPA in 7 d.

b References 5 and 6.

c Reference 7.
